# Vitamin D, intermediary metabolism and prostate cancer tumor progression

**DOI:** 10.3389/fphys.2014.00183

**Published:** 2014-05-15

**Authors:** Wei-Lin W. Wang, Martin Tenniswood

**Affiliations:** Department of Biomedical Sciences, University at Albany, State University of New YorkAlbany, NY, USA

**Keywords:** vitamin D, androgen, prostate, warburg, miRNA, mRNA

## Abstract

Epidemiological data have demonstrated an inverse association between serum vitamin D_3_ levels, cancer incidence and related mortality. However, the effects of vitamin D on prostate cancer biology and its utility for prevention of prostate cancer progression are not as well-defined. The data are often conflicting: some reports suggest that vitamin D_3_ induces apoptosis in androgen dependent prostate cancer cell lines, while others suggest that vitamin D_3_ only induces cell cycle arrest. Recent molecular studies have identified an extensive synergistic crosstalk between the vitamin D- and androgen-mediated mRNA and miRNA expression, adding an additional layer of post-transcriptional regulation to the known VDR- and AR-regulated gene activation. The Warburg effect, the inefficient metabolic pathway that converts glucose to lactate for rapid energy generation, is a phenomenon common to many different types of cancer. This process supports cell proliferation and promotes cancer progression via alteration of glucose, glutamine and lipid metabolism. Prostate cancer is a notable exception to this general process since the metabolic switch that occurs early during malignancy is the reverse of the Warburg effect. This “anti-Warburg effect” is due to the unique biology of normal prostate cells that harbor a truncated TCA cycle that is required to produce and secret citrate. In prostate cancer cells, the TCA cycle activity is restored and citrate oxidation is used to produce energy for cancer cell proliferation. 1,25(OH)_2_D_3_ and androgen together modulates the TCA cycle via transcriptional regulation of zinc transporters, suggesting that 1,25(OH)_2_D_3_ and androgen maintain normal prostate metabolism by blocking citrate oxidation. These data demonstrate the importance of androgens in the anti-proliferative effect of vitamin D in prostate cancer and highlight the importance of understanding the crosstalk between these two signaling pathways.

## Overview on prostate cancer biology

**Figure 1:**
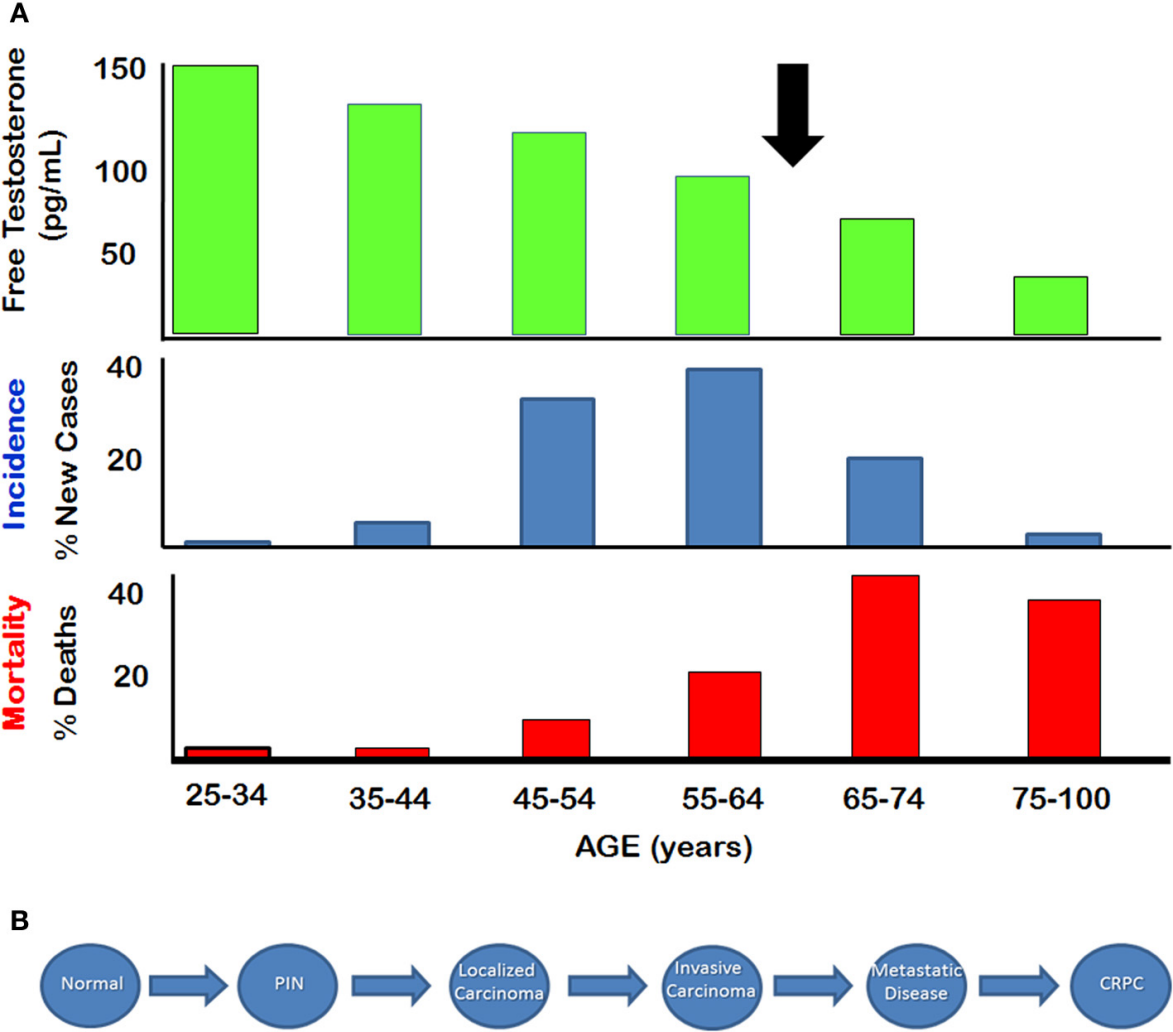
**Natural History of Prostate Cancer (A) Relationship between serum free testosterone and incidence and mortality of prostate cancer**. Arrow indicates approximate age at which free testosterone declines below 50% of the level seen in young adults. **(B)** Stepwise depiction of progression from normal disease to metastatic disease. Castration resistant prostate cancer (CRPC) appears to emerge after hormone therapy. (Figure adapted from SEER database and from Framington Heart Study).

Prostate cancer is the most commonly diagnosed non-cutaneous malignancy in males in North America (Altekruse et al., 2010). This disease is usually considered to be an androgen dependent cancer, since the normal prostate is clearly dependent on androgens for its structure and function. Paradoxically, the age-dependent incidence and associated mortality of prostate cancer between 50 and 60 years of age increase after serum testosterone levels start to decline significantly, particularly after age of 65 (Figure 1A) (Siegel et al., 2014). Prostate adenocarcinomas are slow growing tumors that are characterized by low mitotic index and a long natural history (McNeal, 1968). The progression from normal prostate to prostatic intraepithelial neoplasia (PIN), and eventually to localized adenocarcinoma takes place over several decades (Figure 1B). Autopsy studies have shown that prostatic adenocarcinoma and the pre-malignant PIN are evident in men in their early and mid-thirties. The development of advanced, locally invasive prostate cancer and metastatic disease is a relatively late process for which there are limited treatments, and hormone ablation therapy used at this late stage applies selective stress that probably is responsible for the development of castration-resistant prostate cancer (CRPC).

## Vitamin D and prostate

There are many epidemiological studies that suggest high serum vitamin D levels, usually measured as serum 25(OH)-vitamin D_3_ (25(OH)D_3_) may be important in preventing various cancers, including breast, ovarian and colon cancer (Thorne and Campbell, 2008; Giovannucci, 2009). The risk of developing and dying of these cancers appears to be inversely correlated with sun exposure, and/or vitamin D status, suggesting that vitamin D has chemopreventive properties (Garland et al., 2009). Some studies have also suggested that vitamin D may play a role in prostate cancer prevention (Tseng et al., 2004; Schwartz and Skinner, 2007), but the data are less conclusive than in other cancers and several recent meta-analyses have found weak or no associations between 25(OH)D_3_ levels and prostate tumor incidence or progression (Yin et al., 2009; van der et al., 2009; Barnett et al., 2010; Park et al., 2010; Holt et al., 2013). However, a recent study of men diagnosed with prostate cancer showed that 72% of men with recurrent disease and 68% with clinically localized disease were insufficient or deficient in serum 25(OH)D_3_ levels, less than 20 ng/mL (desirable levels >40 ng/mL) (Trump et al., 2010). These data suggest that the majority of men with prostate cancer have low circulating androgen and low 25(OH)D_3_ levels at the time of diagnosis. Based on many *in vitro* studies (Miller, 1998; Blutt et al., 2000; Peehl et al., 2003), preclinical and clinical studies (Deeb et al., 2007), it has been suggested that vitamin D can be used either as chemopreventative or as therapeutic agent for prostate cancer. Despite extensive research, the importance of vitamin D as a chemopreventive agent for prostate cancer is still a matter of considerable controversy (van der et al., 2009; Park et al., 2010), and the results from therapeutic intervention using 1,25-dihydroxyvitamin D_3_ (1,25(OH)_2_D_3_), the active metabolite of vitamin D_3_ or its less calcemic analogs, have been generally disappointing (Vijayakumar et al., 2005; Beer and Myrthue, 2006; Wagner et al., 2013). In low-risk prostate cancer patients who enrolled in active surveillance, high dose of vitamin D_3_ supplementation decreases Gleason score or the number of positive cores in more than 50% of patient population (Marshall et al., 2012), whereas 1,25(OH)_2_D_3_ supplementation at adjuvant settings have provided mixed results in CRPC or recurrent diseases (Flaig et al., 2006; Chan et al., 2008; Srinivas and Feldman, 2009; Chadha et al., 2010; Scher et al., 2011; Shamseddine et al., 2013).

Various reports suggest that the action of vitamin D in prostate cancer cells is androgen dependent (Esquenet et al., 1995; Murthy et al., 2005; Weigel, 2007; Mordan-McCombs et al., 2010). In Sprague–Dawley rats, 1,25(OH)_2_D_3_ administration decreases prostatic size in intact males, but not castrated groups (Leman et al., 2003). Longitudinal studies have demonstrated a positive correlation between 25(OH)D_3_ levels and the production of androgen (Wehr et al., 2010; Pilz et al., 2011; Nimptsch et al., 2012), which has been further validated *in vitro* (Lundqvist et al., 2011). However, vitamin D also induces *CYP3A4* and *CYP3A5* expression, enzymes that metabolize and inactivate testosterone and androstanediol in prostate cells, suggesting that vitamin D signaling may play a role in limiting androgen levels in the prostate (Maguire et al., 2012). Previous *in vitro* studies have shown that 1,25(OH)_2_D_3_ also induces moderate increases in *AR*, *PSA*, and *TMPRSS2* transcript levels (Hsieh et al., 1996; Zhao et al., 1999; Krishnan et al., 2004; Washington and Weigel, 2010), however this finding does not translate into clinical setting where 1,25(OH)_2_D_3_ appears to decrease the PSA velocity (Krishnan et al., 2003). Based on these findings, serum vitamin D levels appear to have a significant impact on androgen-mediated signaling and the crosstalk between androgen and vitamin D probably plays an important role in prostate cancer biology. While there have been many studies examining the effects of androgens or 1,25(OH)_2_D_3_ individually on gene expression in prostate cancer cells, there have been very few studies that explored the crosstalk between the two signaling pathways and the biological consequences of this crosstalk.

## Genomic overlay of VDR and AR signaling

**Figure 2:**
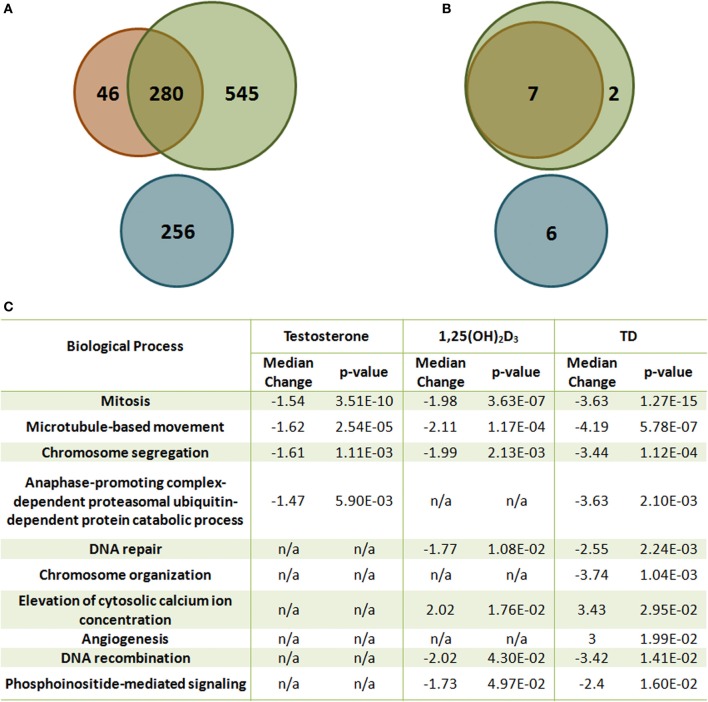
**The effect of T and 1,25(OH)_2_D_3_ on mRNA and miRNA expression in LNCaP cells. (A)** Venn diagram analysis of the gene expression microarray data [Green: 1,25(OH)_2_D_3_-moduated, Orange: androgen-modulated, Blue: synergistically modulated genes by androgen and 1,25(OH)_2_D_3_]. **(B)** Venn diagram analysis of the miRNA microarray data [Green: 1,25(OH)_2_D_3_-modulated, Orange: androgen-modulated, Blue: synergistically modulated genes by androgen and 1,25(OH)_2_D_3_]. **(C)** Gene set enrichment analysis of representative gene sets identified as significantly enriched after 1,25(OH)_2_D_3_ treatment in the presence or absence of androgen in LNCaP cells. False discovery rate <5%.

The crosstalk between VDR- and AR-mediated gene expression was first demonstrated in LNCaP cells (Qiao and Tuohimaa, 2004). Induction of *FACL3* (long-chain fatty-acid CoA ligase 3) is dependent on both vitamin D and androgen levels, and treatment with bicalutamide inhibits 1,25(OH)_2_D_3_-induced *FACL3* expression. This coordinated effect on gene expression has recently been validated by a comprehensive microarray study using the same *in vitro* model (Wang et al., 2011). 1,25(OH)_2_D_3_ and androgen share many common targets and coordinately modulate these transcript levels in the same direction (Figures 2A,B). More importantly, the combination of the two hormones regulates additional genes, including both mRNAs and miRNAs that have not been previously identified. The significance of this additional layer of transcriptional control is best illustrated by bioinformatic analysis which demonstrates the coordinated effect of 1,25(OH)_2_D_3_ and androgen on cellular processes, including cell homeostasis, proliferation, differentiation and metabolism, all of which have significant impact on prostate tumorigenesis (Figure 2C). Most of these processes are more significantly regulated by 1,25(OH)_2_D_3_ and androgen together than by either hormone alone, demonstrating the interaction between the two signaling pathways. Several genes identified from the expression microarray analysis are validated VDR and AR targets, contains functional VDRE (within 10kb upstream and 5 kb downstream of the structural gene) and ARE sites, and some genes exhibit additive induction after testosterone and 1,25(OH)_2_D_3_ stimulation. Both androgen and 1,25(OH)_2_D_3_ induces *PSA* mRNA levels while addition of testosterone blunts the early vitamin D dependent induction of *Cyp24A1*, the main enzyme involved in the catabolism of 1,25(OH)_2_D_3_. This suggests that the half-life of 1,25(OH)_2_D_3_ is extended in the presence of exogenous androgen.

More than 50% of the responsive genes found from microarray data lack functional response elements in their promoters when comparing to existing genome-wide screens for VDREs and androgen responsive database (Wang et al., 2005; Jiang et al., 2009), raising issues regarding the regulation of these genes, particularly genes that are only expressed if both hormones are present.

The anti-neoplastic effect of vitamin D has been linked to its regulation of miRNA levels. This include the repression of *miR-181ab* expression (Wang et al., 2009) and the induction of *miR-100*, *miR-125b*, and *miR-22* levels by 1,25(OH)_2_D_3_ (Alvarez-Diaz et al., 2012; Giangreco et al., 2013). Dysregulated miR-106b expression, which is required for the 1,25(OH)_2_D_3_-induced feed-forward loop regulating *p21* expression in non-malignant RWPE-1 cells, has also been implicated in prostate cancer biology (Poliseno et al., 2010; Thorne et al., 2011). Microarray analysis that interrogates the differential miRNA expression in LNCaP cells after treatment with 1,25(OH)_2_D_3_ and testosterone, either alone or in combination suggests that VDR plays a critical role in miRNA regulation (Wang et al., 2011) and further highlights the important interactions between VDR- and AR-mediated miRNA expression. These include the additive induction of *miR-22*, *miR-29ab*, *miR-134*, *miR-371-5p*, *miR-663*, and *miR-1207-5p* and the synergistic down-regulation of the oncogenic *miR-17/92* cluster by testosterone and 1,25(OH)_2_D_3_. Both *miR-22* and members of the *miR-29* family are candidate tumor suppressors (Alvarez-Diaz et al., 2012; Szczyrba et al., 2013; Wu et al., 2013) and their induction is consistent with the anti-proliferative effect of vitamin D in prostate cancer. In comparison, elevated *miR-371-5p* and *miR-663* expression have been correlated with cancer progression and *miR-663* expression positively associates with the Gleason score used to stage prostate cancer (Zhou et al., 2012; Liu et al., 2013; Jiao et al., 2014). In contrast, the *miR-17/92* cluster is known to play an oncogenic role and its expression has been linked to more advanced prostate cancer (He et al., 2005; Volinia et al., 2006; Sylvestre et al., 2007; Yu et al., 2008; Diosdado et al., 2009; Trompeter et al., 2011; Yang et al., 2013). In addition, this cluster is a well-validated target for c-Myc, which itself is a direct target of VDR (Simpson et al., 1987; O'Donnell et al., 2005), and a recent report has proposed a regulatory role for the *miR-17/92* cluster on PPARα levels, linking *miR-17/92* to energy metabolism in prostate cancer cells (Wang et al., 2013). This concurrent analysis of VDR- and AR-mediated mRNA and miRNA expression reveals an extensive and complex transcription network that interconnects c-Myc, PPARα and other transcription factor-mediated signaling, which is only active when both androgen and vitamin D are present. A recent comprehensive analysis of 24 nuclear receptors and 14 transcription factors (TFs) in the MCF-7 breast cancer cell line has demonstrated a similar finding and has identified genomic regions enriched with nuclear receptors and TFs binding sites, which generates extensive regulatory networks that may modulate target gene expression (Kittler et al., 2013). Such functional interactions between nuclear receptors and TFs, including the antagonistic interaction between RARs and AR and PPARδ (Rivera-Gonzalez et al., 2012; Kittler et al., 2013), and the agonistic interaction between VDR and AR (Wang et al., 2011) provide valuable information that can be used to improve cancer prevention and therapy. The functional interactions between AR and VDR, as well as other nuclear receptors and TFs may also be important for disease management, especially now that nutritional intervention has become more widely accepted as an effective approach to prevent cancer progression. These experimental data suggest that 1,25(OH)_2_D_3_, and androgens as well as other hormones and growth factors trigger at least three mechanisms to modulate global gene expression. These include AR- and VDR-mediated gene transactivation; miRNA-mediated mRNA degradation and translational repression; and transcription factor-mediated feed-forward signaling. These mechanisms do not appear to be mutually exclusive and act together to regulate many vitamin D- and androgen-mediated cellular processes that have significant implication in prostate carcinogenesis.

## Intermediate metabolism: the Warburg effect

A number of studies have suggested that vitamin D has a novel role in regulating energy metabolism. The vitamin D receptor knockout (VDRKO) and the *Cyp27b1* knockout (Cyp27b1KO) mice exhibit elevated energy expenditure with subsequent loss of body fat over time (Narvaez et al., 2009; Wong et al., 2011). In human adipocytes, 1,25(OH)_2_D_3_ inhibits uncoupling protein-1 expression and alters Ca^2+^ homeostasis, suggesting a regulatory role of vitamin D in thermogenesis and provides rationale for the observed lean phenotype in VDRKO and Cyp27b1KO mice (Xue et al., 1998; Shi et al., 2001, 2002). Similarly, both 25(OH)D_3_ and 1,25(OH)_2_D_3_ promotes lipogenesis in primary human preadipocytes, adipocyte and adipose-derived mesenchymal progenitor cells, which is associated with increased expression of differentiation markers *C/EBPα* and *PPARγ* (Nimitphong et al., 2012; Narvaez et al., 2013). However, this effect may be cell type and lineage specific since 1,25(OH)_2_D_3_ inhibits lipid accumulation in mouse 3T3-L1 preadipocytes and prevents high fat diet-induced fatty liver syndrome in Sprague–Dawley male rats (Rayalam et al., 2008; Yin et al., 2012).

In T47D breast cancer cells, 1,25(OH)_2_D_3_ induces lipid synthesis, which has been associated with its effect on cell differentiation and reduced cell growth (Lazzaro et al., 2000). This lipogenic effect of 1,25(OH)_2_D_3_ is recapitulated in LNCaP cells and is enhanced in the presence of androgen (Esquenet et al., 1997; Wang et al., 2013), highlighting the coordinated effect of AR and VDR signaling. Increase in PPARα expression and its associated lipogenic gene signature, including the elevation of fatty acid synthase (*FASN*) expression, accounts for vitamin D- and androgen-induced lipid production. However, this occurs without significant changes in nuclear sterol regulatory element-binding protein (SREBP-1) levels. Nuclear activation of SREBP-1 has been implicated in *de novo* lipogenesis in more aggressive cancers, including prostate cancer (Menendez and Lupu, 2007; Huang et al., 2012). A recent comprehensive parallel analysis of various genomic studies using prostate cancer cell lines has uncovered a critical regulatory role of AR in the energy metabolic network, with lipid synthesis being the predominate AR-regulated process. These data suggest that altered AR signaling and its effects on the downstream targets of calcium/calmodulin-dependent protein kinase kinase 2, beta (*CAMKK2*), which regulates the activity of a key energy sensor AMP-activated protein kinase (AMPK), promotes the metabolic switch that provides the energy for prostate cancer growth and progression (Massie et al., 2011). These data suggest a divergent role of lipid production in prostate tumors: SREBP-1 dependent up-regulation of fatty acids production for phospholipid and membrane synthesis and signaling molecules that are essential for tumor progression (Currie et al., 2013; Soga, 2013); or SREBP-1-independent elevation of neutral and inactive lipid accumulation which restricts energy expenditure and limits tumor growth.

In addition to the modulation of lipid metabolism by vitamin D and androgen, qPCR analysis has suggested a regulatory role of these two hormones on the TCA cycle in prostate cancer cells. In most normal cells, the TCA cycle is utilized to generate energy for normal cellular functions. This process is relatively slow and ATP production does not meet the demand for highly proliferative cancer cells. As a result, cancer cells often disengage mitochondrial oxidative phosphorylation from glycolysis for rapid ATP production by employing the fermentation process, a process referred as the Warburg effect (Warburg et al., 1927; Warburg, 1956; Soga, 2013). Prostate cancer cells are a notable exception, and the metabolic switch that occurs is more appropriately regarded as an “anti-Warburg” effect. The prostate gland normally secrets high levels of citrate into the seminal fluid, a function that is supported by a truncated TCA cycle activity. The prostate has the highest levels of intracellular zinc of any tissue in the body. This high level of zinc inactivates m-aconitase 2 activity, the enzyme that converts citrate to isocitrate in the mitochondria. In prostate cancer cells, zinc transporters are down-regulated, which leads to lower intracellular zinc levels. This restores m-aconitase 2 function and the conversion of citrate to isocitrate for ATP production via the TCA cycle (Costello and Franklin, 1991a,b). This is supported by both clinical and *in vitro* data, demonstrating a minimal reliance of prostate cancer cells on glycolysis for proliferation especially during the early phases of tumor progression. This precludes the usage of fluorine-18-labeled 2-deoxy-2-fluoro-_D_-glucose (FDG-PET) for prostate cancer detection and diagnosis (Hofer et al., 1999; Jadvar, 2011). In comparison, androgen stimulates glucose usage to facilitate citrate accumulation in normal prostate epithelial cells (Harkonen, 1981; Harkonen et al., 1982) and this androgenic effect is maintained in androgen responsive prostate cancer cells, although in these cells, elevated citrate is funneled for the production of lipid (Moon et al., 2011).

**Figure 3:**
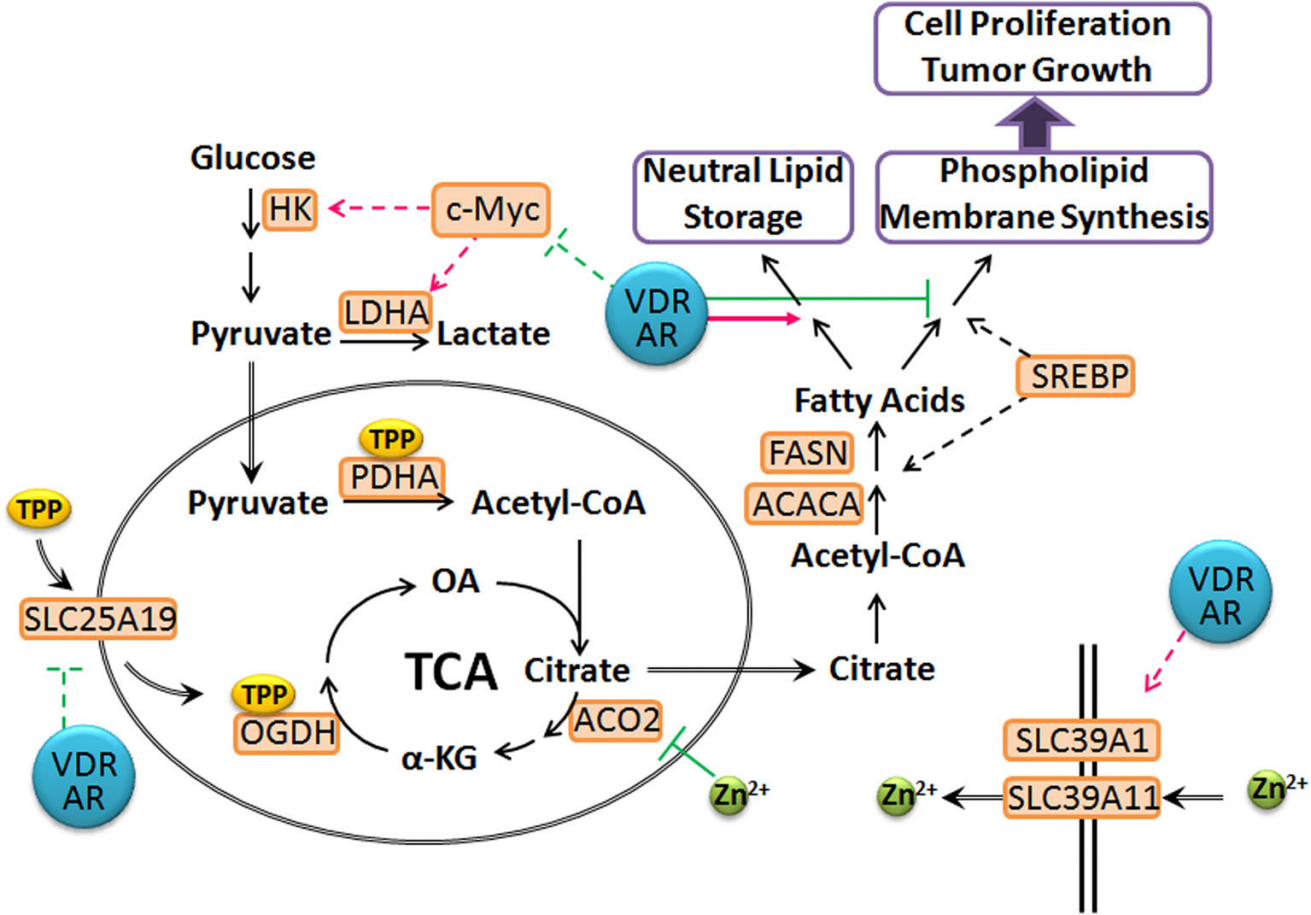
**Proposed model of T- and 1,25(OH)_2_D_3_-mediated prostate cancer metabolism**. The VDR and AR axes modulate the expression of SLC25A19 and SLC39A11, leading to elevated intracellular zinc levels and deplete mitochondrial TPP pool, resulting in a truncated TCA cycle. Citrate is shunted into lipid synthesis and storage instead of phospholipid and membrane synthesis, which prevents cancer cell proliferation. In addition, VDR and AR repress c-Myc levels and associated metabolic reprogramming to maintain prostate cancer cells in a more differentiated state. (dashed line: transcriptional regulation; green: inhibition; magenta: stimulation).

In LNCaP cells, 1,25(OH)_2_D_3_ and androgen together down-regulate mitochondrial thiamine pyrophosphate (TPP) carrier (*SLC25A19*) and up-regulates two zinc transporters, (*SLC39A1* and *SLC39A11*) (supplemental data to Wang et al., 2011). Low expression of *SLC39A1* in adenocarcinomatous glands and PIN foci has been documented and linked to depleted zinc levels (Franklin et al., 2005). In comparison, *SLC39A11* is less well-characterized, but studies have shown that it is abundantly expressed in murine testes and digestive system, and is associated with zinc import (Yu et al., 2013). This suggests that vitamin D and androgen cooperate to reset zinc levels, inhibiting m-aconitase activity in prostate cancer cells. In comparison, down-regulation of the TPP carrier, *SLC25A19* (Lindhurst et al., 2006; Kang and Samuels, 2008) affects mitochondrial coenzyme TPP levels, leading to decreased activities of pyruvate dehydrogenase (PDH) and alpha-ketoglutarate dehydrogenase (OGDH) activities. In comparison, *in vivo* studies have shown that administration of testosterone up-regulates the expression and activities of PDH and mitochondrial aspartate aminotransferase to increase the substrate pools for citrate synthesis, acetyl-CoA and oxaloacetate (Costello and Franklin, 1993; Qian et al., 1993). This suggests that vitamin D and androgen supplementation facilitate the reversion of the metabolic switch that occurs during prostate carcinogenesis by preventing citrate oxidation, partially restoring the normal prostatic function and shunting citrate into the cytoplasm for secretion and lipid synthesis (Figure 3). This is supported by the observation that LNCaP cells retain the sensitivity to androgen-induced citrate production and accumulation (Franklin et al., 1995). This suggests that vitamin D facilitates and maintains this differentiated phenotype, rendering prostate cancer cells less aggressive. This also suggests that maintaining or restoring adequate levels of androgen, accompanied by vitamin D supplement will significantly delay prostate cancer progression in aging men.

To further highlight the impact of vitamin D and androgen on resetting cancer cell metabolism, 1,25(OH)_2_D_3_ and androgen also down-regulate c-Myc levels, whose many functions include metabolic reprogramming to drive tumor progression, including the induction of glycolysis and glutaminolysis (Shim et al., 1998; Wise et al., 2008; Soga, 2013; Zirath et al., 2013). While there is good evidence suggesting a positive correlation between serum glutamate levels and more aggressive prostate cancer (Koochekpour et al., 2012), the dependence of prostate cancer on glutaminolysis for energy generation and progression is not well-studied. Nevertheless, it is reasonable to suggest that in response to vitamin D and androgen stimulation, prostate cancer cells reverse or block the metabolic switch that occurs early in the course of the disease and further blocks c-Myc-mediated metabolic reprogramming, which may occur independently of the initial metabolic switch.

## Conclusion

Recent studies have shown a complex relationship between vitamin D_3_- and androgen-mediated signaling in the normal prostate and prostate cancer through their coordinated effect on mRNA and miRNA transcription, cell proliferation and cancer metabolism. These data suggest that the effect of vitamin D_3_ on global gene expression is dependent on the activity of androgen and their combined effect on miRNA transcription and other TFs. Phenotypically, the two hormones maintain normal prostatic metabolism to prevent de-differentiation of prostate cancer cells into more aggressive phenotype. These newly emerging data provide a explanation for the discrepancies observed from epidemiological and experimental studies of vitamin D_3_ in prostate cancer since these studies do not take the synergistic interactions between the two pathways into account. These data also suggest that maintenance of adequate levels of vitamin D_3_ and androgen will slow or halt prostate cancer progression especially for patients diagnosed with early stage, locally confined disease. Case-control clinical studies will be needed to fully evaluate the risk and benefit of combining these two hormones in prostate cancer patients.

### Conflict of interest statement

The authors declare that the research was conducted in the absence of any commercial or financial relationships that could be construed as a potential conflict of interest.
